# Machine Anomalous Sound Detection Method Based on Lightweight Temporal Pyramid and ECA-MobileFaceNet

**DOI:** 10.3390/s26103214

**Published:** 2026-05-19

**Authors:** Yuezhou Wu, Xiaogen Ye, Qiang Fu, Wenan Zhang

**Affiliations:** 1 School of Computer Science and Artificial Intelligence, Civil Aviation Flight University of China, Guanghan 618307, China; csfuqiang@cafuc.edu.cn (Q.F.); zwa_cafuc@163.com (W.Z.); 2 Flight Technology and Flight Safety Key Laboratory of CAAC, Guanghan 618307, China

**Keywords:** anomalous sound detection, equipment failure, ECA mechanism, multi-scale temporal modeling, MobileFaceNet

## Abstract

To address the challenges of scarce anomaly samples, inadequate modeling of temporal dynamic features, and limited feature selection capability of lightweight models in industrial anomalous sound detection, this paper proposes a method under an unsupervised framework. In the time-domain feature extraction branch, a Lightweight Temporal Pyramid Module (LTPM) is introduced to enhance the multi-scale temporal modeling capability of TgramNet, capturing both short-term and long-term temporal dependencies. In the classification network, the Efficient Channel Attention (ECA) mechanism is embedded into the bottleneck structure of MobileFaceNet to enable adaptive channel recalibration. Furthermore, three waveform-level data augmentation strategies—noise perturbation, time shifting, and amplitude scaling—are adopted. Experimental results on the DCASE 2020 Task 2 dataset demonstrate that the proposed method achieves competitive performance compared with existing approaches, attaining optimal or highly competitive results across multiple machine types. The minimum Area Under the Curve (mAUC) across different machine IDs, along with ROC curve analysis, verifies the stability and generalization capability of the model. This method offers a promising lightweight approach for industrial anomalous sound detection and condition monitoring applications.

## 1. Introduction

Industrial equipment is rapidly evolving toward intelligence and autonomy [1,2,3,4,5,6,7,8,9,10,11,12,13,14,15,16,17,18,19,20,21,22,23]. Reliable condition monitoring plays a critical role in ensuring safe system operation, improving production efficiency, and reducing maintenance costs [4]. In this context, acoustic signals have gradually become an important information carrier in industrial monitoring due to their advantages such as non-invasiveness, low deployment cost, and high sensitivity to mechanical anomalies. Compared with traditional contact-based monitoring methods, audio-based monitoring can capture potential fault features without direct contact with the equipment, offering significant application value in complex or inaccessible industrial environments. Anomalous Sound Detection (ASD) [5] aims to automatically identify potential faults by analyzing acoustic differences between normal and abnormal equipment states, combining signal processing and machine learning methods. Relevant tasks in the DCASE challenge have further promoted the research and application of ASD in the industrial domain [6], attracting widespread attention from both academia and industry. Specifically, acoustic sensors such as microphone arrays can detect subtle changes in equipment operating sounds caused by faults—including contamination, leakage, rotating unbalance, shaft bending, and gear deformation [7,8]—making them a valuable tool for early fault detection and condition monitoring in practical industrial environments.

**Figure 1:**
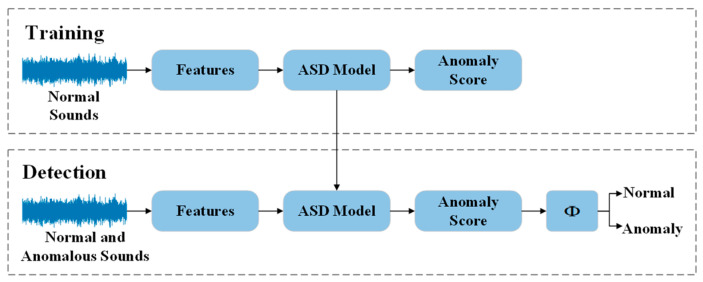
Framework of the unsupervised anomalous sound detection system. The ASD model denotes the neural network used for anomalous sound detection. During the training phase, the model is trained using only normal data. φ represents the threshold. When the anomaly score exceeds this threshold, the sound is classified as anomalous.

Despite the engineering significance of ASD, it still faces numerous challenges in real-world industrial scenarios. Among them, the most critical issue is the scarcity and diversity of anomalous samples [9]. In actual production environments, equipment typically operates under normal conditions for extended periods, while abnormal events occur infrequently and exhibit diverse forms, making it difficult to obtain sufficient and representative anomalous data [10]. In this context, unsupervised learning has gradually become the dominant paradigm in ASD research. As illustrated in Figure 1, in a typical unsupervised framework, the model is trained using only normal sound data. During the testing phase, it discriminates based on a predefined threshold applied to the anomaly score: when the anomaly score exceeds the threshold, the sample is classified as anomalous; otherwise, it is classified as normal.

Traditional unsupervised ASD methods employ autoencoders (AEs) and their variants [11,12,13,14,15,16,17], such as the interpolated deep neural network (IDNN) [11], ID-conditioned autoencoder [12], and variational autoencoder (VAE) [17]. These methods learn the data distribution by minimizing the reconstruction error of normal samples and perform discrimination based on the assumption that anomalous samples yield larger reconstruction errors. However, because the model may learn a general representation that fits both normal and anomalous samples, such methods often suffer from insufficient discriminative ability and a tendency to overfit.

To improve detection performance, researchers have begun to make improvements from the perspective of acoustic feature representation. The Log-Mel spectrogram has been widely adopted in ASD tasks due to its conformity with the human auditory perception mechanism [18,19]. However, its non-uniform frequency distribution based on the Mel filter bank [20] results in low resolution in the high-frequency region, potentially leading to the loss of critical anomaly information. Furthermore, the short-time Fourier transform (STFT) inherently involves a trade-off between time resolution and frequency resolution, which to some extent limits the model’s ability to characterize complex acoustic patterns. To compensate for the limitations of spectral features, some studies have introduced time-domain modeling methods (e.g., TgramNet) [21] to enhance the representation of temporal dependencies, yet their characterization of local temporal dynamic patterns remains insufficient.

In terms of model architecture, early methods were primarily based on reconstruction or density estimation strategies, such as IDNN [10] or normalizing flow models [22,23]. These methods have improved detection performance to some extent but still face challenges such as limited generalization ability or high computational overhead. In recent years, discriminative deep models have gradually become the mainstream direction, among which lightweight networks such as the MobileNet series [24,25,26,27,28] offer advantages for practical industrial deployment due to their low parameter count and computational complexity. However, existing research has mostly focused on improvements in network architecture or training strategies, while targeted enhancements of feature extraction capability remain insufficient. Meanwhile, although attention mechanisms have been introduced to enhance the representation of critical features [29], related methods typically rely on a single feature input and still inadequately model temporal information.

Based on the above analysis, it can be seen that current ASD methods still suffer from two main limitations: first, limited capability in modeling fine-grained temporal dynamic features in audio signals; second, the balance between critical feature selection and representational capacity in lightweight models still leaves room for improvement. To address these issues, this paper enhances the feature extraction and representation capabilities of the model by introducing a time-domain feature modeling module and an attention mechanism. Specifically, a Lightweight Temporal Pyramid Module (LTPM) is embedded into the feature extraction pipeline to strengthen the characterization of local temporal patterns. Meanwhile, an Efficient Channel Attention (ECA) mechanism is introduced into the network architecture to improve the model’s ability to select critical features. Furthermore, by incorporating several simple yet effective audio data augmentation strategies (e.g., noise perturbation, time shifting, and amplitude scaling), the model’s robustness in complex environments is further enhanced.

The main contributions of this study are as follows:

- A lightweight temporal pyramid module (LTPM) is constructed by integrating dilated convolutions and residual fusion, effectively enhancing temporal feature representation for ASD tasks with limited computational overhead.
- The Efficient Channel Attention (ECA) mechanism is integrated into a lightweight network architecture to improve the model’s ability to select critical features with low computational overhead.
- In addition, several commonly used waveform-level augmentation strategies are incorporated during training to improve robustness under complex acoustic environments.
- Experimental validation on the DCASE 2020 Task 2 dataset demonstrates that the proposed method achieves consistent performance improvements in terms of AUC and pAUC metrics.

## 2. Methods

### 2.1. Log-Mel

To transform raw audio into a perceptually meaningful representation, this work employs the Log-Mel spectrogram. This two-dimensional, time–frequency feature effectively embodies the non-linear human auditory perception [30]. The computation begins by partitioning a raw one-dimensional mono audio signal, $x\in R^{1\times L}$, into a sequence of overlapping frames. Each frame is weighted using a Hanning window, and a Short-Time Fourier Transform (STFT) is subsequently applied. For an audio sample x[m], this frequency decomposition is formally given by:

(1) $$X\left[n,k\right]=\sum_{m=n-\left(N_{W}-1\right)}^{n}\;x\left[m\right]\omega \left[n-m\right]e^{-j2\pi km/N}$$

where *n* indexes the temporal frame, $N_{W}$ is the window length; ω[·] denotes the windowing function, k is the frequency bin index, and N is the FFT size. This process yields a complex spectrogram from which the power spectrum, ${\left|STFT(x)\right|}^{2}$, is derived.

The linear frequency axis of the resulting spectrum is then warped to the Mel scale, formulated as:

(2) $$mel\left(f\right)=2595\log_{10}\left(1+\frac{f}{700}\right)$$

This mapping is implemented by a bank of 128 triangular filters, designated as $H_{M}$, which performs a weighted summation of the power spectrum’s frequency bins. The final Log-Mel spectrogram $F_{LM}\in R^{M\times T}$ is obtained by applying a logarithmic compression to the filter bank’s output:

(3) $$F_{LM}=\log\left(H_{M}\cdot |STFT\left(x\right){|}^{2}\right)$$

### 2.2. TgramNet

**Figure 2:**
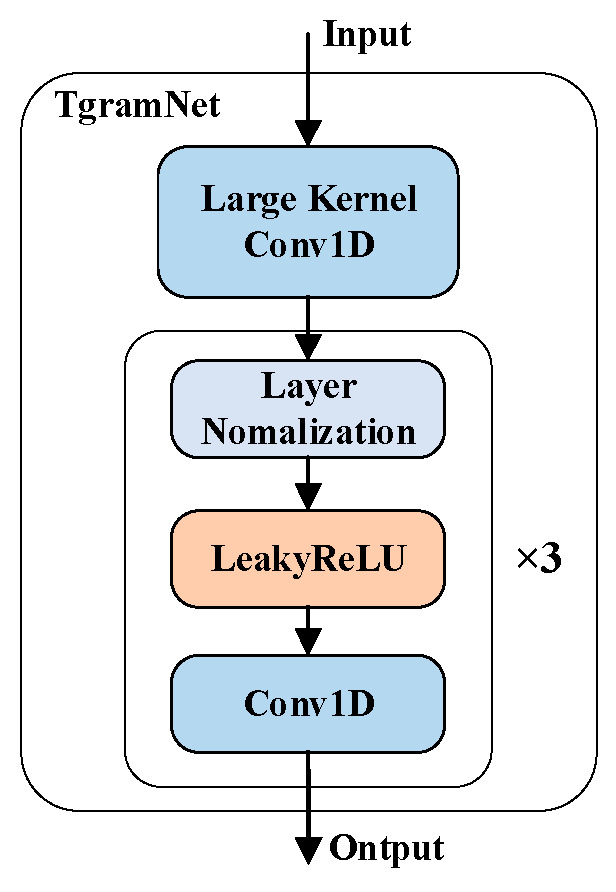
Architecture of TgramNet.

To directly model temporal dynamics from the waveform, a dedicated feature extractor, TgramNet, is employed. This design draws inspiration from the convolutional interpretation of the STFT, where a large-kernel 1D convolutional layer initializes the process, conceptually mirroring a set of learnable bandpass filters [21]. Following this initial decomposition, the network refines the representation through multiple convolutional blocks to capture temporal dependencies. This architectural concept is illustrated in Figure 2, and its configuration is detailed in Table 1.

As implemented in our model, the first layer is a 1D convolution (nn.Conv1d) configured with an input channel of 1, an output channel of 128 (corresponding to the number of Mel bins, mel_bins), a kernel size of 1024 (win_len), and a stride of 512 (hop_len). This configuration directly isolates a time–frequency-like decomposition from the raw audio. After the initialization, a sequence of three identical blocks is applied. Each block is composed of a Layer Normalization (nn.LayerNorm) and a LeakyReLU activation, followed by a second 1D convolution (nn.Conv1d). This latter convolution is configured with 128 input and output channels, a kernel size of 3, a stride of 1, and a padding of 1. This structural design preserves the temporal resolution and dimensionality throughout the network, ensuring the final output retains a precise temporal structure. The computation of the resulting time-domain feature, denoted by $F_{T}$, is defined as:

(4) $$F_{T}=TN\left(x\right)$$

where TN(⋅) represents the complete TgramNet transformation. The output $F_{T}\in R^{M\times T}$ maintains a dimensionality of 128 × 313 units, which is strictly congruent with the Log-Mel spectrogram, facilitating direct feature-level concatenation in subsequent stages of the model.

### 2.3. ArcFace

The Softmax function is extensively employed in image classification tasks, as it transforms the outputs of a classification problem into a probability distribution, thereby distinguishing features of different categories [31]. Nevertheless, Softmax does not impose explicit constraints on intra-class compactness or inter-class separability, which can limit its effectiveness in achieving high classification accuracy.

The Softmax function is defined as follows:

(5) $$L_{s}=-\frac{1}{m}\sum_{i=1}^{\infty }\;ln\left(\frac{e^{W_{\gamma_{i}}^{T}x_{i}+b_{\gamma_{i}}}}{\sum_{j=1}^{n}\;e^{W_{j}^{T}x_{i}+b_{j}}}\right)$$

In this expression, m denotes the batch size; i indexes the spectrogram; j represents the true label and $W_{y_{i}}^{T}x_{i}+b_{y_{i}}$ corresponds to the output of the fully connected layer. Here, $W_{y_{i}}^{T}$, $x_{i}$, $b_{y_{i}}$ and $y_{i}$ are the weight vector for the i-th spectrogram class, the feature vector of the spectrogram, the bias for the class, and the ground-truth label, respectively.

**Figure 3:**
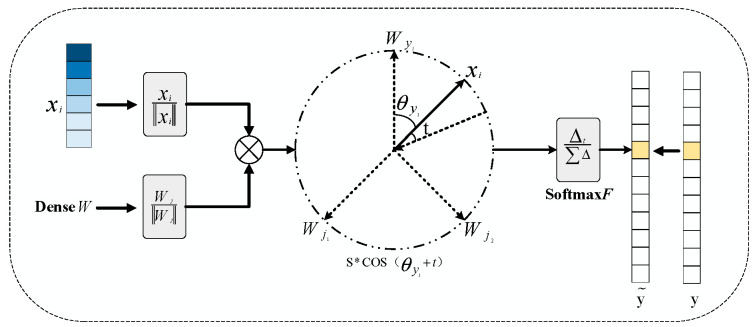
Schematic diagram of the ArcFace Loss classification architecture.

Softmax focuses solely on the correctness of sample classification and lacks mechanisms to enforce intra-class compactness or inter-class discrimination in multi-class tasks. To address these limitations, ArcFace Loss has been proposed as an enhancement over Softmax [32]. By introducing an additive angular margin t(t>0) to the angle θ between $x_{i}$ and $W_{y_{i}}$, an additional penalty is applied to the angle between deep features and their corresponding weights. This approach enhances intra-class compactness while increasing inter-class separability. To facilitate learning of more discriminative angular features, the feature weights are fixed and the bias is set to zero, simplifying the model parameters. A schematic of the ArcFace Loss classification framework is shown in Figure 3.

The ArcFace Loss is defined as:

(6) $$L_{AreFace}=-\frac{1}{m}\sum_{i=1}^{m}\;ln\left(\frac{e^{s\left(cos\left(\theta_{y_{i}}+t\right)\right)}}{e^{s\left(cos\left(\theta_{y_{i}}+t\right)\right)}+\sum_{j=1,j\neq y_{i}}^{N}\;e^{s\cos\theta_{j}}}\right)$$

## 3. Network Model Structure

### 3.1. Model Structure

**Figure 4:**
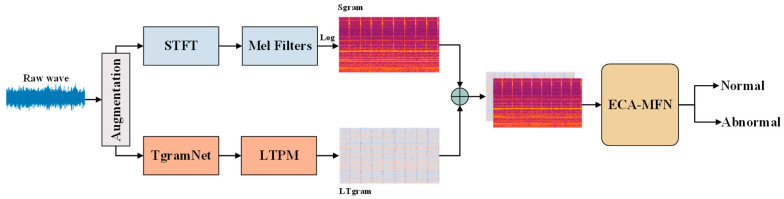
The architecture of the proposed method for ASD.

Figure 4 illustrates the overall framework of the proposed ASD method. Temporal features are extracted from the raw audio through the time-domain branch, where TgramNet is enhanced by the proposed Lightweight Temporal Pyramid Module (LTPM) to capture multi-scale temporal dependencies. Meanwhile, spectral representations are extracted from the Log-Mel spectrogram and encoded via a convolutional backbone network. To improve feature representation capability, we adopt an ECA-enhanced MobileFaceNet (ECA-MFN) as the classifier, enabling adaptive channel-wise recalibration with low computational overhead. Furthermore, we introduce three data augmentation strategies—additive noise, time shifting, and amplitude scaling—to enhance robustness against real-world acoustic variations. Finally, we employ the ArcFace loss function to introduce an angular margin for optimizing the embedding space, thereby significantly improving discriminative capability under different machine conditions.

### 3.2. Data Augmentation

**Figure 5:**
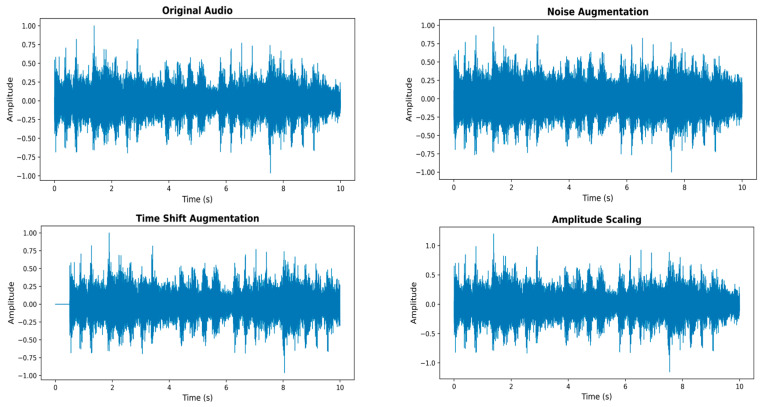
Original audio waveform and augmented audio waveforms.

To improve the model’s generalization capability and reduce overfitting under limited anomalous data conditions, we adopt three waveform-level data augmentation strategies during training, including additive noise perturbation, time shifting, and amplitude scaling. These augmentations are applied exclusively during training and are not used in the testing or evaluation stages. Illustrative examples are shown in Figure 5. For each input audio sample, each augmentation operation is applied with a probability of 0.3.

Specifically, we introduce additive Gaussian noise to the raw waveform, where the noise standard deviation is randomly sampled from a uniform distribution within the range of [0.001, 0.005], simulating common background interference in industrial environments. Time shifting is performed by randomly shifting the waveform along the time axis, with the shift ratio randomly sampled from the range of [0.03, 0.08] of the total signal length. This operation enhances the model’s invariance to temporal misalignment. Amplitude scaling is further achieved by multiplying the waveform by a random factor drawn from the interval [0.9, 1.1], aiming to improve robustness against variations in recording intensity.

### 3.3. LTPM

Existing multi-scale temporal modeling approaches typically increase receptive fields through deep stacking or computationally expensive hierarchical architectures. Although such strategies can capture long-range temporal dependencies, they are often unsuitable for lightweight ASD systems due to increased parameter complexity and inference overhead.

**Figure 6:**
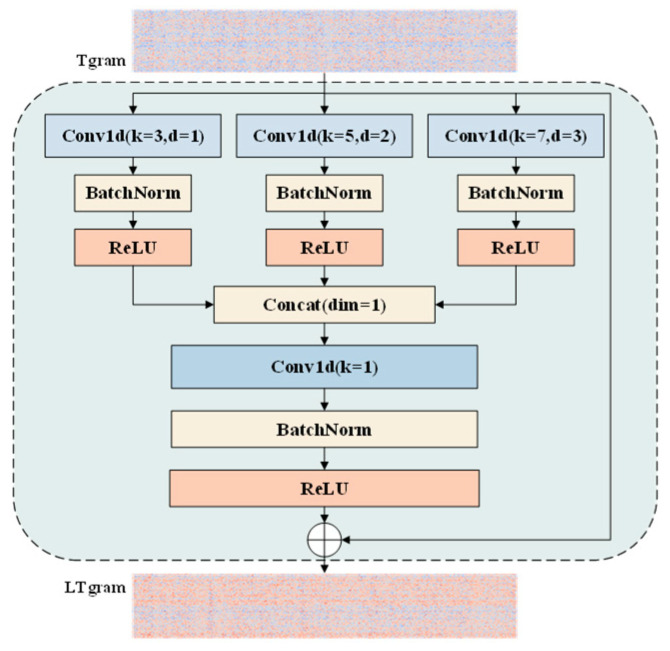
Lightweight parallel temporal pyramid structure of the proposed LTPM for efficient multi-scale acoustic temporal modeling.

Unlike conventional temporal pyramid or deep temporal convolution frameworks, the introduced LTPM is a lightweight parallel temporal modeling module tailored for ASD tasks. It combines shallow multi-branch dilated convolutions with residual fusion to enhance temporal representation while maintaining low computational cost and compatibility with lightweight backbone networks. The module is built upon established components—including dilated convolutions, multi-branch architectures, and residual connections—and its primary contribution lies in the effective lightweight integration and optimization of these techniques for ASD-oriented temporal modeling. Its architecture is illustrated in Figure 6.

Given an input temporal feature map $X\in R^{B\times C\times T}$, where B, C and T denote the batch size, channel dimension, and temporal length, respectively, the LTPM adopts a multi-branch architecture to extract hierarchical temporal features. Specifically, three parallel one-dimensional convolutional branches are constructed, each with different kernel sizes and dilation rates to model temporal patterns under different receptive fields. The first branch employs a standard convolution with a kernel size of 3 and a dilation rate of 1 to capture local temporal dependencies. The second branch uses a kernel size of 5 and a dilation rate of 2, effectively enlarging the receptive field to model mid-range temporal correlations. The third branch further extends the receptive field with a kernel size of 7 and a dilation rate of 3, enabling the extraction of long-range temporal information. Each branch is followed by batch normalization and a ReLU activation function to improve training stability and non-linear representational capability.

The outputs of the three branches are concatenated along the channel dimension to form a unified multi-scale representation. To fuse these features and restore the original channel dimension, a pointwise convolution (i.e., 1×1 convolution) is applied, followed by batch normalization and a ReLU activation. This fusion operation effectively integrates multi-scale temporal information while controlling computational overhead. To further stabilize training and preserve the original temporal information, a residual connection is introduced, which directly adds the input feature map to the fused output. Consequently, the final output of the LTPM can be expressed as:

(7) $$Y={Conv}_{1\times 1}\left(\left[F_{1},F_{2},F_{3}\right]\right)+X$$

where $\left[\cdot \right]$ denotes channel-wise concatenation, and $F_{1},$ $F_{2},$ $F_{3}$ represent the outputs of the three temporal branches, respectively.

By combining multi-scale dilated convolutions and residual learning, the LTPM effectively enhances temporal feature representation without significantly increasing the computational burden. Compared with traditional single-scale temporal modeling, this module can capture both short-term transient characteristics and long-term temporal dependencies, which is crucial for distinguishing between normal and anomalous acoustic patterns. Overall, the LTPM is not proposed as a conceptually novel temporal modeling paradigm, but rather as a carefully optimized lightweight integration of established multi-scale and residual learning techniques tailored for efficient ASD applications.

### 3.4. ECA-MobileFaceNet

The classification backbone network adopted in this study is an improved MobileFaceNet (MFN), which has demonstrated strong capability in feature representation for anomalous sound detection tasks. Compared with traditional convolutional neural networks, MFN employs a lightweight architecture based on inverted residual bottleneck blocks and replaces the global average pooling layer with global depthwise separable convolution (GDConv), enabling the model to better retain discriminative information distributed spatially. To adapt MFN to time–frequency acoustic representations, the network input is formed by concatenating the Log-Mel spectrogram and temporal features, resulting in a dual-channel input. The modified network architecture is shown in Table 2. The overall architecture begins with a standard convolutional layer and a depthwise separable convolutional layer for initial feature extraction. Subsequently, a series of bottleneck blocks are used to progressively capture high-level representations. Each bottleneck block contains a pointwise convolution for channel expansion, a depthwise separable convolution for spatial filtering, and a linear pointwise projection. Residual connections are applied when the input and output dimensions are consistent.

**Figure 7:**
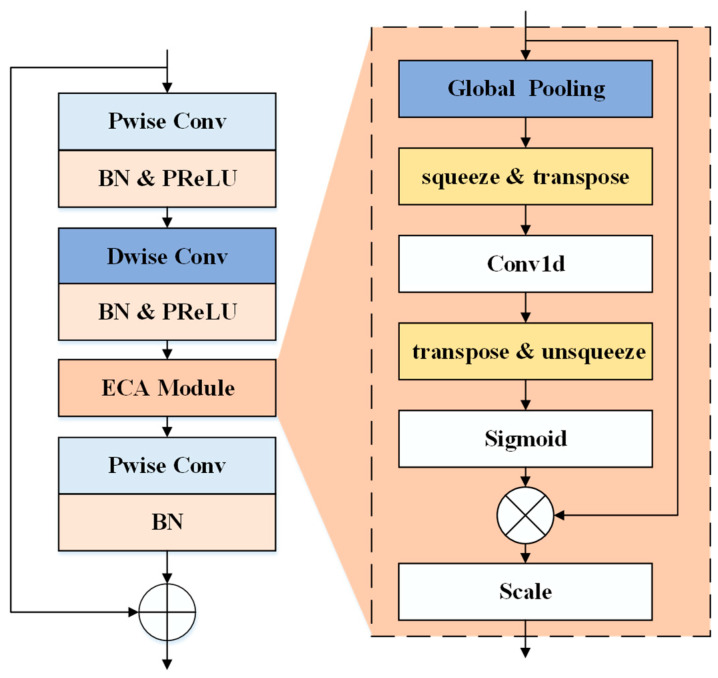
Network architecture of the ECA-based bottleneck module.

To further enhance the channel-level feature representation capability, an Efficient Channel Attention (ECA) module [33] is integrated into each bottleneck block, as illustrated in Figure 7. Specifically, after the depthwise convolution, global spatial information is first aggregated via global average pooling to generate a compact channel descriptor. Unlike the Squeeze-and-Excitation mechanism, the ECA module avoids dimensionality reduction and instead applies a lightweight one-dimensional convolution with an adaptive kernel size to capture local cross-channel interactions. The resulting attention weights are then normalized using a sigmoid function and applied to the feature map through channel-wise multiplication.

Formally, given an intermediate feature map $U\in R^{C\times H\times W}$, the channel descriptor is obtained as follows:

(8) $$z_{c}=\frac{1}{H\times W}\sum_{i=1}^{H}\;\sum_{j=1}^{W}\;U_{c}(i,j)$$

Subsequently, the channel attention weights are computed using a one-dimensional convolution:

(9) s=σ(Conv1D(z))

where σ(∙) denotes the sigmoid function. The refined feature map is then obtained as:

(10) $${\tilde{U}}_{c}=s_{c}\cdot U_{c}$$

By embedding the ECA module into the bottleneck structure, the network can efficiently highlight informative channels while suppressing less relevant responses with low computational cost. After feature extraction through multiple bottleneck stages, a 1×1 convolutional layer is applied to expand the channel dimension to 512. Then, global depthwise separable convolution (GDConv) is employed to aggregate global spatial information, where the kernel size matches the spatial resolution of the feature map. Finally, a linear projection layer is used to reduce the feature dimension to 128, which is then fed into the classification head for anomaly detection. Compared with the original MobileFaceNet designed for face recognition, the proposed architecture is significantly simplified and optimized for acoustic signal processing. The number of bottleneck stages is reduced, and the network is capable of handling time–frequency inputs, resulting in a more efficient and effective backbone network for anomalous sound detection.

## 4. Experimental Setups

### 4.1. Training Details

We train our model on the training set using raw audio signals with a duration of approximately 10 s. The frame size is 1024 samples, the overlap rate for Log-Mel spectrogram computation is 50%, and the number of Mel filter banks is 128 (i.e., W = 1024, H = 512, M = 128). Consequently, the obtained Sgram and LTgram have dimensions of 128 × 313. The Adam optimizer [34] is adopted for model training with a learning rate of 0.0001, and a cosine annealing strategy is used for learning rate decay. The model is trained for 300 epochs with a batch size of 128. The cosine margin and scale parameters of ArcFace [17] are set to 0.7 and 30, respectively. The negative log probability is used as the anomaly score for detection.

The environment used in our experiments is shown in Table 3.

### 4.2. Dataset

In this study, experiments are conducted on the DCASE 2020 Task 2 dataset, which is a widely adopted benchmark for unsupervised anomalous sound detection (ASD). Following the official task configuration, the dataset combines subsets from the MIMII and ToyADMOS datasets to evaluate ASD performance under diverse acoustic and machine operating conditions [6]. In our experiments, we adopt the training data from both the development dataset and the additional dataset provided by the task. The training set consists exclusively of normal operating sound samples from various machine types. The test set, drawn from the development dataset, contains both normal and anomalous sound samples.

**Figure 8:**
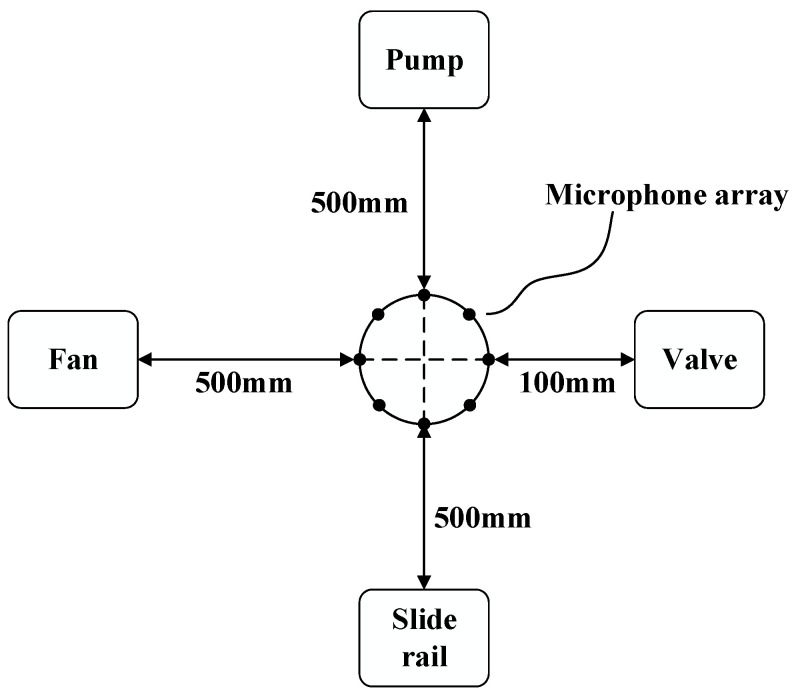
Schematic diagram of the experimental setup for MIMII dataset acquisition.

The MIMII subset contains recordings of real industrial machines, including valves, pumps, fans, and slide rails, collected for anomalous sound detection research [7]. As shown in the data acquisition setup diagram from Reference [7] (Figure 8), the recordings were acquired using a TAMAGO-03 circular microphone array (System In Frontier Inc., Tokyo, Japan) consisting of eight microphones positioned approximately 50 cm from the target machines (10 cm for valves). All recordings were stored as 16-bit audio at a 16 kHz sampling rate under reverberant acoustic conditions. To approximate practical industrial acoustic environments, the recorded machine sounds were additionally mixed with background noise collected from real factory environments. Anomalous sounds were generated by introducing physical faults such as contamination, leakage, rotating unbalance, rail damage, and inadequate lubrication [7].

**Figure 9:**
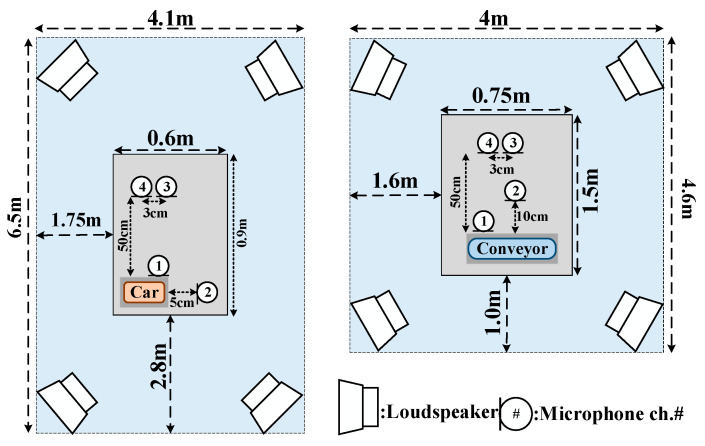
ToyADMOS data acquisition diagram.

The ToyADMOS subset provides operating sounds of miniature mechanical systems recorded under controlled laboratory conditions [8], including the ToyCar and ToyConveyor categories used in this study. As shown in the data acquisition setup diagram from Reference [8] (Figure 9), the recording system employed four omnidirectional SHURE SM11-CN dynamic microphones arranged around the target miniature machines. Environmental noise recorded from actual factory locations (e.g., collision, drilling, and airbrushing sounds) was separately mixed to introduce representative environmental noise conditions during ASD evaluation. Anomalous sounds included bent shafts, deformed or melted gears, foreign objects coiled around tires, and chipped wheel axles [8]. The ToyADMOS dataset provides controllable acoustic conditions, well-defined fault patterns, and standardized recording configurations, which make it suitable as a benchmark for evaluating ASD methods under controlled acoustic acquisition conditions and limited anomalous data scenarios.

Although the ToyADMOS subset provides controllable recording conditions for ASD benchmarking, its acoustic characteristics do not completely match those of full-scale industrial machinery. As acknowledged in the original ToyADMOS publication [8], the detailed spectral characteristics of miniature-machine sounds may differ from those of full-scale industrial machinery due to differences in physical scale, structural characteristics, and operating mechanisms. Therefore, in the present study, the ToyADMOS subset is adopted as a standardized benchmark dataset for evaluating the robustness and generalization capability of ASD models, rather than as a direct acoustic substitute for industrial equipment.

Overall, the DCASE 2020 Task 2 dataset constitutes a heterogeneous benchmark that combines real factory recordings with controlled miniature-machine recordings. This combination enables the evaluation of ASD methods under both practical industrial acoustic conditions and controllable laboratory acoustic conditions.

**Figure 10:**
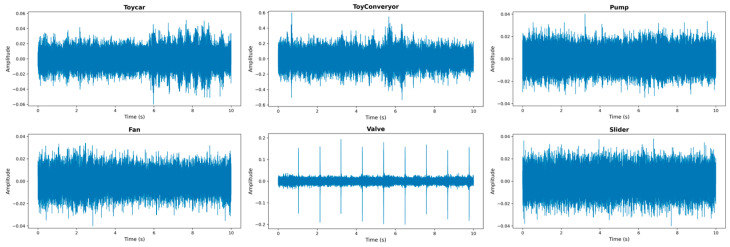
Waveforms of the normal sounds produced by different machines.

The composition of the dataset is shown in Table 4. Figure 10 shows the normal sound waveforms of different machines. As can be seen from the figure, except for the valve, the waveforms of the other machines do not exhibit any obvious regular pattern.

### 4.3. Evaluation Metrics

To conduct a thorough assessment of detection capability, this study adopts three complementary metrics derived from the Receiver Operating Characteristic (ROC) curve: the Area Under the Curve (AUC), the partial AUC (pAUC), and the minimum AUC (mAUC).

The AUC quantifies the aggregate classification performance across all possible detection thresholds, with values closer to 1 signifying stronger overall discriminative power. The pAUC restricts the evaluation scope to a low false-positive-rate (FPR) regime, specifically the interval [0, *p*] where *p* = 0.1, thereby emphasizing the model’s practical utility in scenarios where false alarms must be minimized. The mAUC is defined as the lowest AUC value recorded among individual machine identifiers (IDs) belonging to the same machine type. This metric serves as a conservative estimate of worst-case performance and reflects the model’s stability and robustness against intra-class variability. The formal definitions of these metrics are expressed as follows:

(11) $$AUC=\frac{1}{N_{-}N_{+}}\sum_{i=1}^{N_{-}}\;\sum_{j=1}^{N_{+}}\;H(A(X_{j}^{+})-A(X_{i}^{-}))$$

(12) $$pAUC=\frac{1}{\lfloor pN_{-}\rfloor N_{+}}\sum_{i=1}^{\left\lfloor pN_{-}\right\rfloor }\;\sum_{j=1}^{N_{+}}\;H(A(X_{j}^{+})-A(X_{i}^{-}))$$

(13) $$mAUC=\min_{k\in \kappa }\left(AUC_{k}\right)$$

In the above equations, $N_{+}$ and $N_{-}$ denote the respective counts of anomalous and normal test samples. The function H(x) represents the Heaviside step function, which returns 1 when x>0 and 0 otherwise. The terms $X_{j}^{+}$ and $X_{i}^{-}$ correspond to the learned feature representations of the *j*-th anomalous and *i*-th normal test instances, while $A\left(\cdot \right)$ indicates the anomaly score assigned by the model. For the mAUC calculation, k denotes the set of all machine IDs within a specific machine type, and $AUC_{k}$ is the AUC computed on the test subset corresponding to the *k*-th ID.

## 5. Experimental Results

### 5.1. Performance Comparison

To validate the effectiveness of the proposed method, comparative experiments were conducted with other mainstream acoustic anomalous sound detection methods. The results are shown in Table 5. The results demonstrate that the proposed method achieves the best average performance, with an AUC of 95.22% and a pAUC of 90.43%, outperforming all compared methods. Compared with the baseline model STgram-MFN, the proposed method improves the average AUC from 92.36% to 95.22% and the pAUC from 86.34% to 90.43%, fully demonstrating the effectiveness of the proposed improvements. Compared with the state-of-the-art TASTgram-MFN [35], our method improves the average AUC by 0.95% and the average pAUC by 1.52%.

From a category perspective, the proposed method achieves excellent or highly competitive performance on machine types such as Slider, ToyCar, Pump, and ToyConveyor, outperforming all comparable methods. Although the performance on Valve and Fan is slightly lower than that of the best-performing method, TASTgram-MFN [35], the gap is small and remains within a competitive range. Importantly, the proposed method maintains consistently strong performance across all machine types, indicating superior generalization ability compared to methods that achieve high scores only on specific categories.

To further evaluate the effectiveness of the proposed method under controlled conditions, additional comparative experiments were conducted using identical augmentation settings for STgram-MFN, TASTgram-MFN, and the proposed method. The results are presented in Table 6.

It can be observed that waveform-level augmentation improves the performance of all methods to varying degrees, indicating that data augmentation contributes positively to acoustic anomaly detection performance. For example, the average AUC/pAUC of STgram-MFN increases from 92.36%/86.34% to 93.43%/87.69% after augmentation, while TASTgram-MFN improves from 94.27%/88.91% to 94.40%/89.44%.

Under the same augmentation conditions, the proposed method achieves the best overall performance, reaching 95.22% AUC and 90.43% pAUC. Compared with TASTgram-MFN under identical settings, the proposed method improves the average AUC and pAUC by 0.82% and 0.99%, respectively.

In addition, even without augmentation, the proposed method still achieves competitive performance compared with the baseline STgram-MFN, demonstrating the effectiveness of the proposed temporal modeling and feature enhancement strategy for ASD tasks.

**Figure 11:**
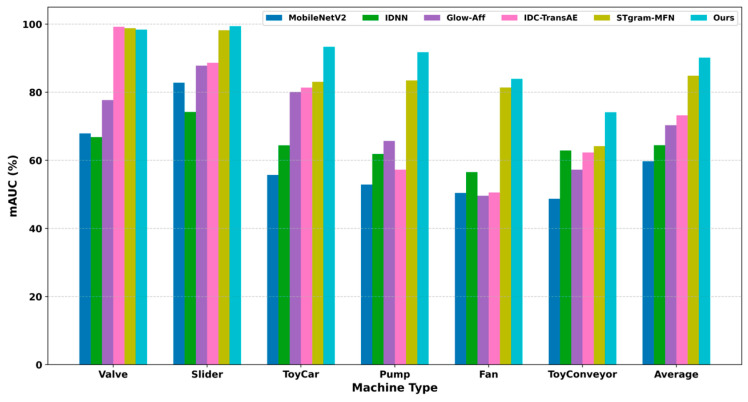
Comparison of mAUC (%) performance across different types of machines.

Furthermore, to further evaluate the stability of the model across different individual machines of the same machine type, this paper adopts the minimum AUC (mAUC) as an evaluation metric, which reflects the detection performance of the model under the most unfavorable conditions. The corresponding experimental results are shown in Figure 11. Compared with existing methods, the proposed method achieves higher mAUC values on multiple machine types such as Slider, ToyCar, Pump, Fan, and ToyConveyor, indicating better generalization ability and robustness across different equipment instances. Particularly in the ToyCar task, the mAUC of the proposed method improves by 10.25% compared to the baseline model STgram-MFN. Overall, the average mAUC achieved by the proposed method reaches 90.14%, which is an increase of 5.28% over STgram-MFN, further demonstrating that the proposed method provides more stable and reliable anomaly detection performance on most machine types.

### 5.2. Ablation Study

To further investigate the contributions of the proposed architectural components and augmentation strategies, a series of controlled ablation experiments were conducted on the DCASE 2020 Task 2 dataset, as summarized in Table 7.

Without data augmentation, introducing only the LTPM module improves the average AUC/pAUC from 92.36%/86.34% to 92.64%/87.28%, while introducing only the ECA module improves the average AUC to 93.09%. When LTPM and ECA are jointly employed, the performance further improves to 93.68%/87.89%, representing gains of 1.32 percentage points in AUC and 1.55 percentage points in pAUC compared with the baseline. These results indicate that the proposed modules enhance temporal feature representation and channel feature discrimination even without augmentation.

After applying data augmentation, the baseline performance increases from 92.36%/86.34% to 93.43%/87.69%, suggesting that augmentation improves model robustness and generalization under limited anomalous data conditions. Incorporating the LTPM module on top of augmentation further improves the performance to 94.69%/89.30%, indicating that lightweight multi-scale temporal modeling remains beneficial under augmented training conditions.

When augmentation, LTPM, and ECA are integrated together, the model achieves the best overall performance with an AUC of 95.22% and a pAUC of 90.43%. Compared with the augmented baseline, the full model achieves additional improvements of 1.79 percentage points in AUC and 2.74 percentage points in pAUC. This result suggests that augmentation, temporal modeling, and channel attention provide complementary benefits for acoustic anomaly detection.

### 5.3. ROC Curve Analysis

**Figure 12:**
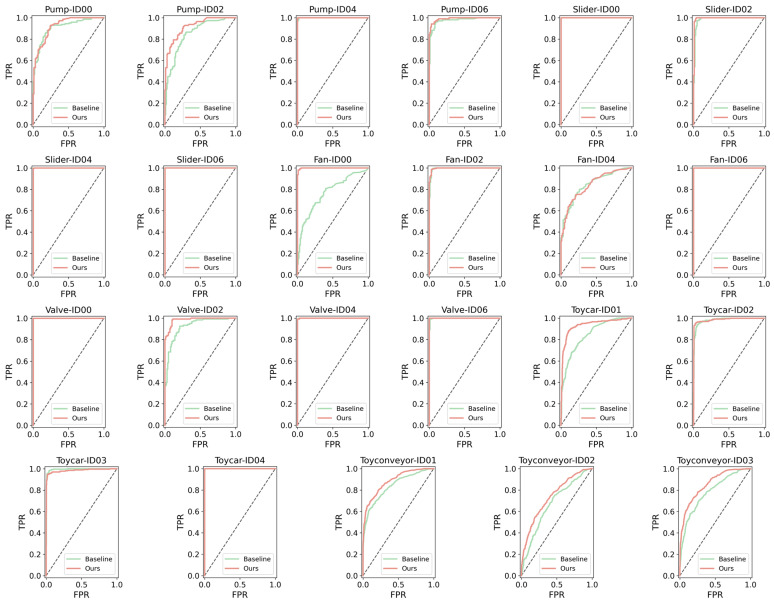
ROC curve comparison.

To further evaluate the stability and discriminative capability of the proposed method, we compared its performance with that of STgram-MFN on the test sets of the six machine types from the DCASE 2020 Challenge Task 2, as shown in Figure 12. Overall, the proposed method achieves superior or competitive detection performance on most machine IDs. Specifically, for the pump, slider, valve, and toyconveyor machine types, the proposed method outperforms STgram-MFN across all corresponding IDs, demonstrating strong consistency and stability. For the fan type, although the performance is slightly lower than that of STgram-MFN on ID04, it achieves higher detection performance on ID00; considering the overall trend, this machine type still exhibits competitive performance. For the toycar type, there is a slight performance drop on a few IDs (e.g., ID03), but the overall difference is small, with limited impact on the overall performance. The above results indicate that the anomaly patterns of different machine types exhibit significant differences in temporal scales and spectral structures. Therefore, effectively modeling both short-term local features and long-term global dependencies in acoustic signals is a key factor affecting anomaly detection performance. The proposed method enhances the model’s ability to represent multi-scale temporal features and critical channel information by introducing a multi-scale temporal modeling module (LTPM) and a lightweight channel attention mechanism (ECA). Meanwhile, combined with data augmentation strategies such as noise, time shifting, and amplitude scaling, the diversity of feature distributions is further increased, endowing the model with stronger discriminative ability and generalization performance under complex operating conditions.

### 5.4. Parameter Count Analysis

Table 8 compares the parameter counts and detection performance of different models. The parameter counts of some compared methods are cited from [41]. Although traditional lightweight models such as IDNN and MobileNetV2 have fewer parameters, they lag significantly behind acoustic feature modeling-based methods in terms of both AUC and pAUC. Compared with TASTgram, the proposed method has a slight increase in parameter count, but further improves the AUC and pAUC to 95.22% and 90.43%, respectively, achieving the best results among all methods in the table. This demonstrates that introducing the LTPM temporal pyramid module, the ECA channel attention mechanism, and data augmentation strategies leads to only a modest increase in parameter count while significantly enhancing the overall detection performance, achieving a favorable balance between model complexity and detection accuracy.

## 6. Conclusions

This paper proposes an effective anomalous sound detection method evaluated on the DCASE 2020 Task 2 benchmark, which encompasses diverse fault types including contamination, leakage, rotating unbalance, rail damage, and deliberate component damage. The framework integrates multi-scale temporal modeling and a lightweight attention mechanism to enhance acoustic feature representation. Specifically, three data augmentation strategies—noise injection, time shifting, and amplitude scaling—are introduced to improve robustness against acoustic variations. In addition, a lightweight temporal feature enhancement module named LTPM is designed to strengthen multi-scale temporal representation for ASD tasks by capturing both short-term and long-term temporal dependencies. Meanwhile, an ECA-based MobileFaceNet is adopted to enhance channel-level feature representation.

Extensive experiments conducted on the DCASE 2020 Task 2 dataset demonstrate that the proposed method achieves performance superior or comparable to several competing methods in terms of both AUC and pAUC. The proposed framework achieves consistent improvements across multiple machine categories and maintains stable detection performance across different machine IDs, particularly in challenging scenarios such as Toy-Conveyor. ROC curve analysis further confirms the stability of the proposed method under diverse benchmark acoustic conditions.

Nevertheless, several limitations should be acknowledged. First, the ablation study indicates that data augmentation contributes substantially to the overall performance improvement, while the architectural enhancements alone provide relatively moderate gains over strong recent baselines such as TASTgram-MFN. Second, for certain machine IDs with highly complex or subtle anomaly patterns, the performance improvement remains limited. Third, the current evaluation is restricted to the publicly available DCASE 2020 Task 2 benchmark, which includes both real industrial machine recordings and controlled miniature-machine recordings. Therefore, the present results should be interpreted primarily as benchmark-level validation of the proposed ASD framework rather than direct verification under practical industrial production environments.

Future work will focus on validating the proposed method using in situ industrial recordings collected under practical operating conditions. In addition, more advanced temporal modeling strategies, self-supervised representation learning methods, and multi-channel acoustic sensing techniques will be explored to further improve robustness and adaptability under challenging industrial acoustic environments. Reducing model complexity while maintaining competitive detection performance also remains an important direction for practical deployment.

## Figures and Tables

**Table 1:** Layer configuration of TgramNet.

Layer	*c*	*k*	*s*	*p*	*n*
Conv1D	128	1024	512	512	×1
LayerNorm	-	-	-	-	×3
Leaky ReLU	-	-	-	-	
Conv1D	128	3	1	1	

**Table 2:** MobileFaceNet network structure.

Operator	*t*	*c*	*s*	*n*
Conv 3 × 3	-	64	1	2
DW Conv 3 × 3	-	64	1	1
Bottleneck	2	128	2	2
Bottleneck	4	128	2	2
Bottleneck	4	128	2	2
Conv 1 × 1	-	512	1	1
GD Conv 8 × 20	-	512	1	1
Conv 1 × 1 (Linear)	-	128	1	1
FC	-	K	1	-

**Table 3:** Experimental Configuration.

Name	Specific Configuration
Operating System	Linux-5.15.0-78-generic-x86_64-with-glibc2.31
Processor	NVIDIA vGPU-32 GB
Memory	32 GB
OS Bit	64-bit
Programming Language	CPython 3.8.10
Dataset	DCASE 2020Task2
Deep Learning Framework	PyTorch 2.4.1

**Table 4:** An overview of DCASE 2020 Challenge Task 2 dataset.

ID	Type	IDs of Machine	Training Samples	Testing Samples
1	ToyCar	7	7000	2459
2	ToyConveyor	6	6000	2509
3	Valve	7	5822	879
4	Pump	7	5766	856
5	Fan	7	6521	1875
6	Slider	7	5174	1290

**Table 5:** Comparison of AUC (%) and pAUC (%) performance across different machine types. Bold and underlined values indicate the best and second-best results, respectively.

Model	Valve		Slider		ToyCar		Pump		Fan		ToyConveyor		Average	
	AUC	pAUC	AUC	pAUC	AUC	pAUC	AUC	pAUC	AUC	pAUC	AUC	pAUC	AUC	pAUC
AutoEncoder [6]	66.28	50.98	84.76	66.53	78.77	67.58	72.89	59.99	65.83	52.45	72.53	60.43	73.51	60.91
IDNN [11]	84.09	64.94	86.45	67.58	78.69	69.22	73.76	61.07	67.71	52.90	71.07	59.70	76.96	63.93
MobileNetV2 [36]	88.65	87.98	95.27	85.22	87.66	85.92	82.53	76.50	80.19	74.40	69.71	56.43	84.00	78.09
Glow-Aff [19]	91.40	75.00	94.60	82.80	92.20	84.10	83.40	73.80	74.90	65.30	71.50	59.00	84.66	74.75
STgram-MFN [21]	99.64	98.44	99.55	97.61	94.44	87.68	91.94	81.75	94.04	88.97	74.57	63.60	92.36	86.34
GeCo [37]	99.06	95.52	98.61	95.26	96.62	89.33	93.09	86.89	92.73	85.19	74.69	68.82	92.47	86.34
SW-WAVENET [38]	99.01	97.26	89.96	94.58	95.49	90.20	91.94	80.68	**97.53**	91.54	81.20	68.20	93.25	87.41
IDC-TransAE [39]	99.60	98.29	96.20	86.38	93.40	87.41	83.41	79.24	80.44	70.21	75.69	62.96	88.12	80.94
CLP-SCF [40]	99.89	99.51	99.57	97.73	95.85	90.19	94.97	87.39	96.98	93.23	75.21	62.79	93.75	88.48
TASTgram-MFN [35]	**99.94**	**99.66**	99.48	97.25	96.32	90.01	95.08	86.28	97.28	**94.16**	77.54	66.09	94.27	88.91
Ours	99.59	98.14	**99.84**	**99.18**	**97.59**	**93.82**	**95.73**	**88.82**	95.86	92.34	**82.69**	**70.27**	**95.22**	**90.43**

**Table 6:** Fair comparison under identical augmentation settings. Bold and underlined values indicate the best and second-best results, respectively.

Model	Augmentation	AUC	pAUC
STgram-MFN	X	92.36	86.34
STgram-MFN	√	93.43	87.69
TASTgram-MFN	X	94.27	88.91
TASTgram-MFN	√	94.40	89.44
Ours	X	93.68	87.89
Ours	√	**95.22**	**90.43**

**Table 7:** Ablation results.

Augmentation	LTPM	ECA	AUC	pAUC
X	X	X	92.36	86.34
√	X	X	93.43	87.69
X	√	X	92.64	87.28
X	X	√	93.09	86.30
X	√	√	93.68	87.89
√	√	X	94.69	89.30
√	√	√	95.22	90.43

**Table 8:** Comparison of parameter count and performance in terms of average AUC (%) and pAUC (%). Bold and underlined values indicate the best and second-best results, respectively.

Method	Parameters	AUC (%)	pAUC (%)
IDNN	**46k**	76.96	62.57
MobileNetV2	1.1M	84.34	77.74
Glow-Aff	30M	85.20	73.90
STgram-MFN	1.1M	92.36	86.34
TASTgram	1.2M	94.27	88.91
Ours	1.48M	**95.22**	**90.43**

## Data Availability

The data used in this paper are publicly available from the DCASE 2020 Challenge Task 2 (Unsupervised Detection of Anomalous Sounds) at https://dcase.community/challenge2020/task-unsupervised-detection-of-anomalous-sounds (accessed on 11 November 2025).

## Abbreviations

The following abbreviations are used in this manuscript:

ASD	Anomalous Sound Detection
Machine ID	Machine identifier
DCASEs	Detection and classification of acoustic scenes and events
Log-Mel	Log-Mel spectrogram
LTPM	Lightweight Temporal Pyramid Module
ECA	Efficient Channel Attention
STFT	Short-time Fourier transform
FFT	Fast Fourier transform
MFN	MobileFaceNet
AUC	Area under the receiver operating characteristic (ROC) curve
pAUC	Partial area under the receiver operating characteristic (ROC) curve
mAUC	Minimum AUC
MIMII	Malfunctioning Industrial Machine Investigation and Inspection (dataset)
ToyADMOS	Toy Acoustic Anomaly Detection in Machine Operating Sounds (dataset)

## References

[1] Thoben K.D., Wiesner S., Wuest T. (2017). “Industrie 4.0” and smart manufacturing-a review of research issues and application examples. Int. J. Autom. Technol..

[2] Zhou J., Li P., Zhou Y., Wang B., Zang J., Meng L. (2018). Toward new-generation intelligent manufacturing. Engineering.

[3] Wang L., Wang G. (2016). Big data in cyber-physical systems, digital manufacturing and industry 4.0. Int. J. Eng. Manuf. (IJEM).

[4] Tran M.Q., Doan H.P., Vu V.Q., Vu L.T. (2023). Machine learning and IoT-based approach for tool condition monitoring: A review and future prospects. Measurement.

[5] Nunes E.C. (2021). Anomalous sound detection with machine learning: A systematic review. arXiv.

[6] Koizumi Y., Kawaguchi Y., Imoto K., Nakamura T., Nikaido Y., Tanabe R., Purohit H., Suefusa K., Endo T., Yasuda M. (2020). Description and discussion on DCASE2020 challenge task2: Unsupervised anomalous sound detection for machine condition monitoring. arXiv.

[7] Purohit H., Tanabe R., Ichige K., Endo T., Nikaido Y., Suefusa K., Kawaguchi Y. (2019). MIMII Dataset: Sound dataset for malfunctioning industrial machine investigation and inspection. arXiv.

[8] Koizumi Y., Saito S., Uematsu H., Harada N., Imoto K. (2019). ToyADMOS: A dataset of miniature-machine operating sounds for anomalous sound detection. Proceedings of the 2019 IEEE Workshop on Applications of Signal Processing to Audio and Acoustics (WASPAA).

[9] Liao J., Yang F., Lu X. (2025). An Enhanced Contrastive Ensemble Learning Method for Anomaly Sound Detection. Appl. Sci..

[10] Nassif A.B., Abu Talib M., Nasir Q., Dakalbab F.M. (2021). Machine learning for anomaly detection: A systematic review. IEEE Access.

[11] Suefusa K., Nishida T., Purohit H., Tanabe R., Endo T., Kawaguchi Y. (2020). Anomalous sound detection based on interpolation deep neural network. Proceedings of the ICASSP 2020–2020 IEEE International Conference on Acoustics, Speech and Signal Processing (ICASSP).

[12] Kapka S. (2020). ID-conditioned auto-encoder for unsupervised anomaly detection. arXiv.

[13] Alam J., Boulianne G., Gupta V., Fathan A. An ensemble approach to unsupervised anomalous sound detection. Proceedings of the 5th Workshop on Detection and Classification of Acoustic Scenes and Events (DCASE).

[14] Daniluk P., Gozdziewski M., Kapka S., Kośmider M. (2020). Ensemble of Auto-Encoder Based Systems for Anomaly Detection. DCASE2020 Challenge. https://dcase.community/documents/challenge2020/technical_reports/DCASE2020_Daniluk_140_t2.pdf.

[15] Hayashi T., Yoshimura T., Adachi Y. (2020). Conformer-Based Id-Aware Autoencoder for Unsupervised Anomalous Sound Detection. DCASE2020 Challenge. https://dcase.community/documents/challenge2020/technical_reports/DCASE2020_Hayashi_111_t2.pdf.

[16] Park J., Sooyeon Y., Yoo S. Unsupervised detection of anomalous machine sound using various spectral features and focused hypothesis test in the reverberant and noisy environment. Proceedings of the Detection and Classification of Acoustic Scenes and Events, DCASE2020.

[17] Zhang C., Wei Y., Wang X., Wu X., Zhu X. (2025). Machine sound anomaly detection based on dual-channel feature fusion variational auto-encoder. Appl. Intell..

[18] Giri R., Tenneti S., Cheng F., Helwani K., Isik U., Krishnaswamy A. (2020). Self-Supervised Classification for Detecting Anomalous Sounds. https://assets.amazon.science/8f/33/04709ab4460da4af7f80528ab61c/self-supervised-classification-for-detecting-anomalous-sounds.pdf.

[19] Dohi K., Endo T., Purohit H., Tanabe R., Kawaguchi Y. (2021). Flow-based self-supervised density estimation for anomalous sound detection. Proceedings of the ICASSP 2021–2021 IEEE International Conference on Acoustics, Speech and Signal Processing (ICASSP).

[20] Davis S., Mermelstein P. (1980). Comparison of parametric representations for monosyllabic word recognition in continuously spoken sentences. IEEE Trans. Acoust. Speech Signal Process..

[21] Liu Y., Guan J., Zhu Q., Wang W. (2022). Anomalous sound detection using spectral-temporal information fusion. Proceedings of the ICASSP 2022–2022 IEEE International Conference on Acoustics, Speech and Signal Processing (ICASSP).

[22] Kingma D.P., Dhariwal P. (2018). Glow: Generative flow with invertible 1 × 1 convolutions. arXiv.

[23] Papamakarios G., Pavlakou T., Murray I. (2017). Masked autoregressive flow for density estimation. arXiv.

[24] Howard A.G., Zhu M., Chen B., Kalenichenko D., Wang W., Weyand T., Andreetto M., Adam H. (2017). Mobilenets: Efficient convolutional neural networks for mobile vision applications. arXiv.

[25] Sandler M., Howard A., Zhu M., Zhmoginov A., Chen L.-C. Mobilenetv2: Inverted residuals and linear bottlenecks. Proceedings of the IEEE Conference on Computer Vision and Pattern Recognition.

[26] Bi Z., Jiang J., Zhang W., Shan M. (2026). Anomalous Sound Detection by Fusing Spectral Enhancement and Frequency-Gated Attention. Mathematics.

[27] Wang M., Mei Q., Song X., Liu X., Kan R., Yao F., Xiong J., Qiu H. (2023). A machine anomalous sound detection method using the lMS spectrogram and ES-MobileNetV3 network. Appl. Sci..

[28] Kong D., Yu H., Yuan G. (2024). Multi-spectral and multi-temporal features fusion with se network for anomalous sound detection. IEEE Access.

[29] Vaswani A., Shazeer N., Parmar N., Uszkoreit J., Jones L., Gomez A.N., Kaiser L., Polosukhin I. (2017). Attention is all you need. arXiv.

[30] Pham L., Phan H., Nguyen T., Palaniappan R., Mertins A., McLoughlin I. (2021). Robust acoustic scene classification using a multi-spectrogram encoder-decoder framework. Digit. Signal Process..

[31] Liu W., Wen Y., Yu Z., Yang M. (2016). Large-margin softmax loss for convolutional neural networks. arXiv.

[32] Deng J., Guo J., Xue N., Zafeiriou S. Arcface: Additive angular margin loss for deep face recognition. Proceedings of the IEEE/CVF Conference on Computer Vision and Pattern Recognition.

[33] Wang Q., Wu B., Zhu P., Li P., Zuo W., Hu Q. ECA-Net: Efficient channel attention for deep convolutional neural networks. Proceedings of the IEEE/CVF Conference on Computer Vision and Pattern Recognition.

[34] Kingma D.P., Ba J. (2014). Adam: A method for stochastic optimization. arXiv.

[35] Choi S., Choi J.W. (2024). Noisy-arcmix: Additive noisy angular margin loss combined with mixup for anomalous sound detection. Proceedings of the ICASSP 2024–2024 IEEE International Conference on Acoustics, Speech and Signal Processing (ICASSP).

[36] Giri R., Tenneti S.V., Helwani K., Helwani K., Isik U., Krishnaswamy A. (2020). Unsupervised Anomalous Sound Detection Using Self-Supervised Classification and Group Masked Autoencoder for Density Estimation. Challenge on Detection and Classification of Acoustic Scenes and Events (DCASE 2020 Challenge). https://dcase.community/documents/challenge2020/technical_reports/DCASE2020_Giri_103_t2.pdf.

[37] Zeng X.M., Song Y., Zhuo Z., Zhou Y., Li Y.H., Xue H., Dai L.R., McLoughlin I. (2023). Joint generative-contrastive representation learning for anomalous sound detection. Proceedings of the ICASSP 2023–2023 IEEE International Conference on Acoustics, Speech and Signal Processing (ICASSP).

[38] Chen H., Ran L., Sun X., Cai C. (2023). SW-WAVENET: Learning representation from spectrogram and wavegram using wavenet for anomalous sound detection. Proceedings of the ICASSP 2023–2023 IEEE International Conference on Acoustics, Speech and Signal Processing (ICASSP).

[39] Guan J., Liu Y., Kong Q., Xiao F., Zhu Q., Tian J., Wang W. (2023). Transformer-based autoencoder with ID constraint for unsupervised anomalous sound detection. EURASIP J. Audio Speech Music. Process..

[40] Guan J., Xiao F., Liu Y., Zhu Q., Wang W. (2023). Anomalous sound detection using audio representation with machine ID based contrastive learning pretraining. Proceedings of the ICASSP 2023–2023 IEEE International Conference on Acoustics, Speech and Signal Processing (ICASSP).

[41] Neri M., Carli M. (2024). Low-complexity attention-based unsupervised anomalous sound detection exploiting separable convolutions and angular loss. IEEE Sens. Lett..

